# Emergence of *Besnoitia besnoiti* in Belgium

**DOI:** 10.3390/pathogens10121529

**Published:** 2021-11-23

**Authors:** Laurent Delooz, Julien Evrard, Serge Eugene Mpouam, Claude Saegerman

**Affiliations:** 1 Regional Association for Animal Registration and Health (ARSIA) ASBL, 5590 Ciney, Belgium; julien.evrard@arsia.be; 2 Research Unit of Epidemiology and Risk Analysis Applied to Veterinary Science (UREAR-ULiège), Fundamental and Applied Research for Animals & Health (FARAH) Center, Faculty of Veterinary Medicine, University of Liege, 4000 Liege, Belgium; 3 School of Veterinary Medicine and Science, University of Ngaoundere, Ngaoundere P.O. Box 454, Cameroon; sempouam@yahoo.fr

**Keywords:** besnoitiosis, *Besnoïtia besnoiti*, introduction, spread, surveillance, cattle, Belgium

## Abstract

Bovine besnoitiosis is a cattle disease caused by a protozoan parasite called *Besnoitia besnoiti.* It is of serious economic concern to the cattle industry and also compromises animal welfare. For several years, it has been considered an emerging disease in some countries and regions located in the north of Europe far away from the known endemic areas in the south. This study describes the situation in the southern part of Belgium, where the parasite was recently introduced through imports of animals coming from departments of France where the disease was present. It details the detection of clinical cases as well as disease transmission features related to contacts during grazing and sales of infected cattle. A tracking and monitoring system was quickly set up and detected twelve outbreaks. Several cattle were controlled, but the lack of appropriate regulations weakens disease-management efforts. Hopefully, this predictable and silent introduction triggers the awareness of decision-makers about the need for an appropriate prevention and control policy, law enforcement, and the implementation of necessary measures to avoid bovine besnoitiosis becoming endemic in Belgium or other non-endemic countries. In addition, more proactive surveillance is required from authorities through threat analysis in the context of the risk of emergence or re-emergence of infectious animal diseases.

## 1. Introduction

Bovine besnoitiosis is a disease affecting cattle. It is caused by *Besnoitia besnoiti* (*B. besnoiti*), a protozoan parasite close to *Toxoplasma gondii* and *Neospora caninum* [1]. There are several recognised and unrecognised species in the genus Besnoiti [2] that affect different animal species. This study focuses exclusively on bovine besnoitiosis due to *B. besnoiti*. Cattle are the only known intermediate host of *B. besnoiti*. The entire life cycle remains unknown [3]. There is evidence that biting insects can mechanically transmit *B. besnoiti* [4,5,6].

This disease presents two distinct phases: a first acute phase and then a chronic phase. Each of these phases includes a series of distinct clinical signs. The clinical signs of the disease are characterised by skin and systemic clinical signs. The latter are very helpful for bovine besnoitiosis surveillance and diagnosis. However, relying only on clinical signs for detection can lead to wrong diagnoses related to other infectious diseases characterised by similar clinical signs. It is therefore essential to carry out confirmatory laboratory tests. Different laboratory diagnostic methods (histopathology, polymerase chain reaction (PCR), the immunofluorescence antibody test (IFAT), Western blot (WB), and enzyme-linked immunosorbent assay (ELISA)) are available for the detection of *B. besnoiti* infection in cattle. The application of these methods or their combination depends on the animal’s and the corresponding herd’s clinical status [3].

Bovine besnoitiosis does not always lead to death but can result in dramatically decreased body condition scores [7] and, in specific cases of pregnant animals, this disease can lead to abortion [8,9]. It compromises animal welfare [10] and is also a serious economic concern to the cattle breeding industry [3].

According to the European Food Safety Authority (EFSA) [10], bovine besnoitiosis should be considered an emerging disease in Europe. At the beginning of the twentieth century, the disease was identified in the southwest of France and in Portugal [11], which have been known to be endemic countries for decades. In Europe, the situation has changed considerably over the last century. Several reasons could explain this new scenario, including animal trade, management practices such as sharing pastures, natural mating, and climate change [12]. Several outbreaks have been reported in Europe in non-endemic countries such as Germany [13], Italy [14], Switzerland [15], Croatia [3], and Hungary [16]. In Belgium, the presence of *B. besnoiti* was first described in 2012 without contamination of other farms. No further reports of suspected *B. besnoiti* infection have been made by farmers and local animal health professionals. The 2012 infection in Belgium was an isolated and imported case from the Pyrenees Mountain region in southern France [17]. As in Italy where more farmers choose French cattle breeds [14], Belgian breeders import animals that are more robust than the Belgian Blue breed, mainly from France. In Belgium, the risk of bovine besnoitiosis introduction is real since thousands of cattle are imported each year from endemic countries or areas such as Portugal, Spain, and southwestern France [18]. As described before, the level of cattle biosecurity implemented in Belgium is relatively low [19]. If *B. besnoiti* emerges in a herd, 10% of the animals are expected to be infected and to lose their commercial value within the following 3 years [20].

**Figure 1:**
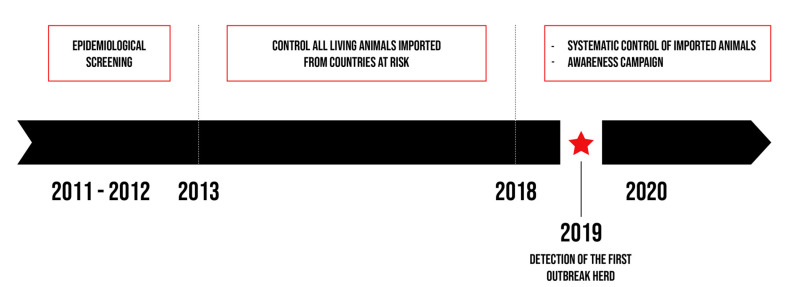
Illustration of the main actions carried out in southern Belgium.

Given the risk associated with importing cattle and the health and economic consequences for cattle breeding, the Regional Association for Animal Registration and Health (ARSIA) performed an epidemiological screening characterised by the systematic control of all live animals imported from endemic countries. It also seemed essential to set up an awareness campaign in order to improve the disease detection in southern Belgium as described in Figure 1 and to take the necessary measures to control bovine besnoitiosis.

Though Belgium remained free of bovine besnoitiosis for several years, the risk of its emergence in the country became real following uncontrolled imports of French infected animals. Indeed, the first outbreak was reported in May 2019. This study describes the evolving disease surveillance in southern Belgium in relation to the introduction and the spread of *B. besnoiti*.

## 2. Results

### 2.1. Active Surveillance

#### 2.1.1. Epidemiological Screening

In December 2012, 5034 samples were collected from 248 selected herds that imported animals from France between February 2011 and November 2012. Those samples were analysed between 2019 and 2021. A total of 29 animals (0.58%; 95% confidence interval (CI): 0.39–0.83) were seropositive for the *B. besnoiti* Ab ELISA. However, all seropositive sera were tested with WB and none of these sera were confirmed to be positive by WB.

#### 2.1.2. Serological Testing of Imported Live Cattle

From January 2018 to July 2021, all animals imported from countries at risk were analysed using *B. besnoiti* Ab ELISA and all seropositive sera were tested with WB. The results of the study are shown in Table 1. This monitoring allowed for the detection of five infected cattle in four newly infected herds.

#### 2.1.3. Control of All Live Animals Imported from Countries at Risk

From May 2019 to July 2021, after the discovery of the first outbreak, ARSIA, aware of the risk of having imported infected animals not yet diagnosed, requested testing of all alive animals not yet tested for besnoitiosis and imported from countries at risk. A total of 3623 bovines in 743 herds were tested using *B. besnoiti* Ab ELISA and eight bovines were seropositive in six herds. All seropositive sera were tested with WB and the infection was confirmed by WB on six animals in four new infected herds (Table 2). Two years after the beginning of this screening, there remained 454 untested animals in 152 herds.

### 2.2. Awareness Campaign and Passive Surveillance

**Figure 2:**
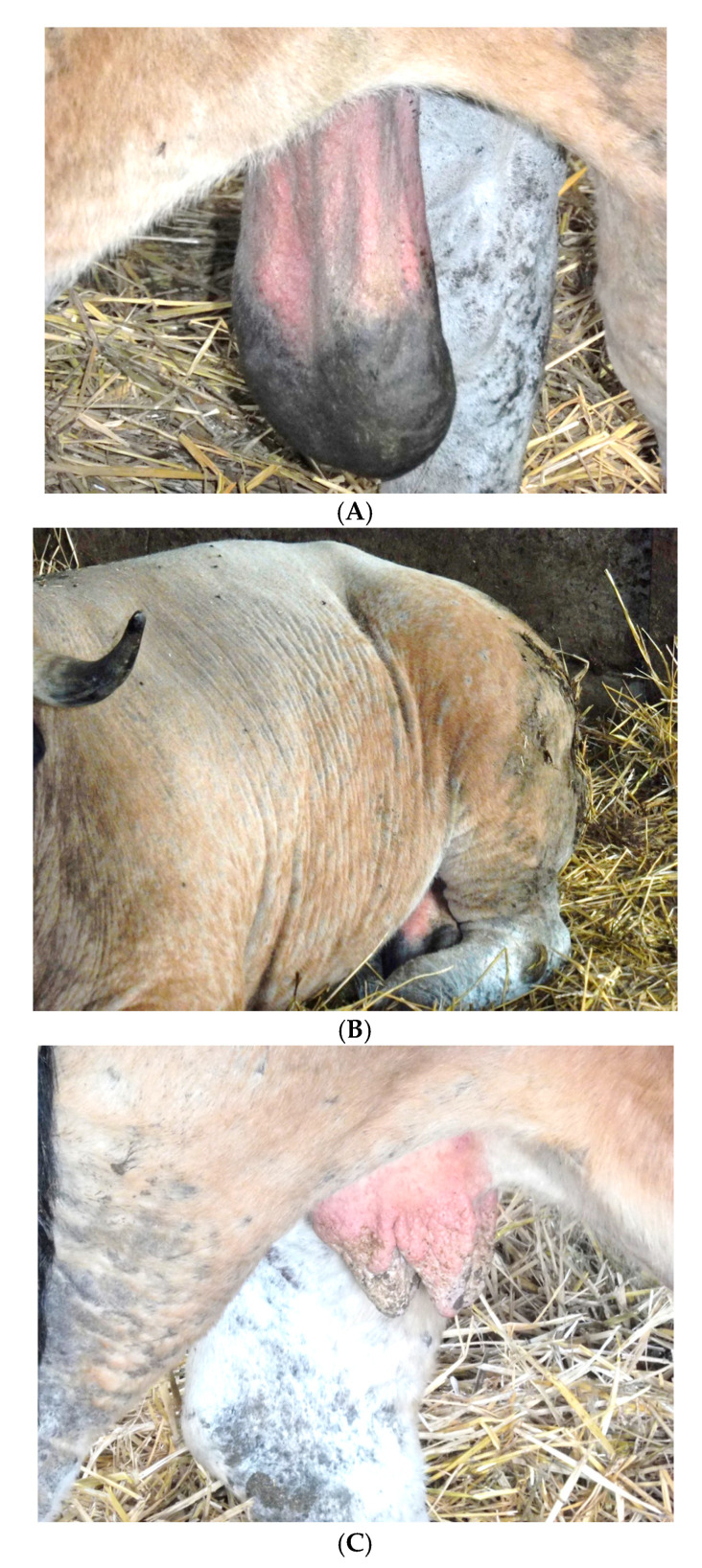
Clinical signs detected on the infected bull: (**A**) Scrotal oedema on the infected bull. Clinical signs detected on the infected cow: (**B**) Elephant skin and alopecia on the 8-year-old infected cow; (**C**) Nodules on the udder and teats of the 8-year-old infected cow.

On 16 May 2019, following the awareness campaign, a veterinary practitioner contacted ARSIA to report clinical suspicions about two cattle from one farm (Herd 1 = H1) raising the Aubrac breed in the province of Liège. The clinical suspicion (Figure 2) on a six-year-old bull imported from the department of Aveyron (France) in February 2015 was related to cutaneous scrotal oedema, scleroderma, lameness and depilation. The clinical signs on an eight-year-old cow born in Belgium of a cow imported from the department of Cantal (France) in March 2010 were major disarmament, cutaneous face oedema, greyish skin with abnormal skin folds at the temples and nodules on the udder and teats (Figure 2). No typical cysts were observed in the eyes or in other body parts of both cases. Serum samples were collected from the two suspicious animals.

Positive *B. besnoiti* Ab ELISA results were obtained for serum collected from both animals. Both sera were confirmed to be positive for *B. besnoiti* by WB. Some days after the confirmation of the diagnosis, 119 animals were present in the herds and a serological screening was performed on all animals older than six months in the herd. In total, 19 (19.6% with 95% CI: 12.2–28.9) animals were seropositive for the *B. besnoiti* Ab ELISA (Table 3). Out of the 19 ELISA positive samples, ten were *B. besnoiti* WB positive.

The repartition of ELISA and WB positives as a function of age stratum (less than two years versus equal to or greater than two years) was not homogenous (Fisher’s exact test; *p*-value = 0.002 and 0.04, respectively). Proportionally more positive animals were aged above two years old than those of two years old or less.

All seropositive animals were slaughtered the same year after diagnosis. The concerned farmer asked for compensation for the infected animals, which delayed the culling process.

Eighteen months after the first diagnostic and six months after the culling of the last infected animal, a second serological screening of all animals older than six months was performed and all 140 cattle tested *B. besnoiti* Ab ELISA negative.

Six months later, a third serological screening of all animals older than 12 months was performed and none of the 63 tested animals were seropositive for the *B. besnoiti* Ab ELISA. Furthermore, no more clinical suspicions of bovine besnoitiosis were reported at this farm after the two last screenings.

### 2.3. Tracing Back

In order to understand the infection’s origin, a tracing back process was performed by contacting GDS France (the French health defense group) and consulting with SANITEL, the Belgian computerised management system for the identification, registration, and monitoring of animals (cattle, sheep, goats, deer, pigs, etc.) (Table 4). According to GDS France, none of the herds where the concerned cattle were bought had a history of bovine besnoitiosis infection. Nevertheless, the departments where the imported infected cattle came from are endemic for besnitoisis (the southern part of France) except for the Doubs department, which is located in the far east of France.

### 2.4. Tracing Forward

A total of seven contact herds in southern Belgium were identified by tracing forward herd H1 by interviewing local veterinary practitioners and farmers about grazing contacts and SANITEL for purchased animals. The tracing forward demonstrated that six contact herds had contact on the pasture and one contact herd purchased animals from H1. Consequently, two herds were infected.

In contact herd 1 (H2), an important grazing contact within 100 m with cattle from herd H1 was identified. A first serological screening was performed on eighteen animals randomly selected and two cattle were *B. besnoiti* Ab ELISA seropositive, which was confirmed by WB. One month later, a serological screening of all animals older than 12 months was performed to complete the first screening and 3.45% (2/58) of the animals were *B. besnoiti* Ab ELISA seropositive and the two seropositive samples were confirmed by WB (Table 5). The seropositive animals were directly slaughtered, although they did not show clinical presentation signs of bovine besnoitiosis.

Six months later, a second serological screening of all animals older than 12 months was performed and none of the 59 tested animals from contact farms were seropositive.

With respect to contact herd 2 (H3), identified following the sale of three cows aged between 2 and 3 years, a serological screening of all animals (N = 3) was performed. One of the three animals, a two-year-old cow, was *B. besnoiti* Ab ELISA seropositive, which was confirmed by WB. The infected cow was slaughtered eight months after the diagnosis. A second serological screening of all animals was performed 6 months later, and the two other cows remained seronegative.

A serological screening was performed in each contact herd (from contact herd 3 to contact herd 7) as described in Table 6. No seropositive animals were detected out of the 20 cattle tested.

Regarding the tracing of the second outbreak herd (H2), 27 contact herds were identified, one in southern Belgium and twenty-six in northern Belgium. All grazing contact herds of H2 were common to H1. The purpose of buying cattle from purchase contacts was breeding and fattening. The tracing carried out in southern Belgium showed that only the herd that bought six animals (contact herd 8) was infected. The Regional Association of Animal Health in Flanders (DGZ) did not communicate the screening results about the 26 contact herds in northern Belgium.

**Figure 3:**
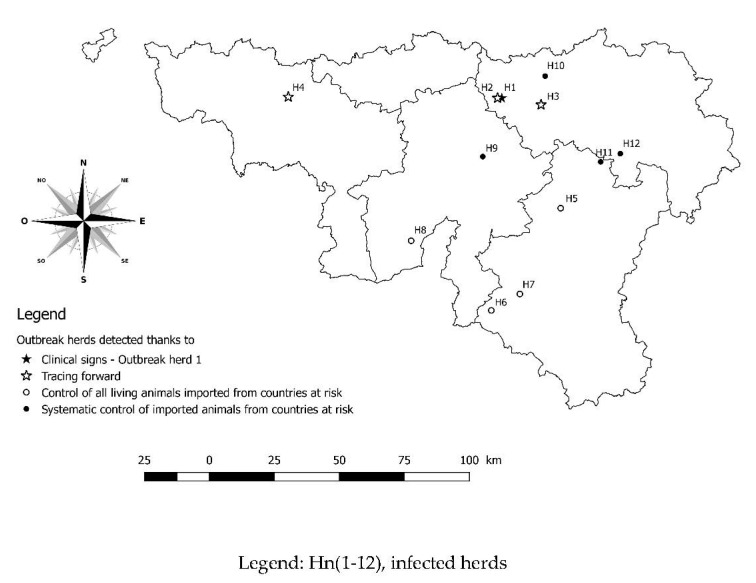
Geographical localisation of all twelve outbreak herds in the southern part of Belgium.

Concerning the other outbreak herds (H5, H6, H7, H8, H9, H10, H11, and H12) located in different provinces of the studied region (Figure 3), the tracing was performed and each herd had one or more seropositive cattle and all seropositive samples were confirmed by WB. In these cases, no clinical signs of bovine besnoitiosis were noticed by local veterinary practitioners. The concerned breeders all had an extensive grazing system.

## 3. Discussion

Until now, besnoitiosis has not been a notifiable disease in Belgium (due to an absence of national and European regulation). An epidemiological status assessment of bovine besnoitiosis was carried out in southern Belgium in 2012. The results obtained in 2012 during that first explorative serological survey through monitoring showed an apparent seroprevalence of zero (95% CI: 0–0.06%). Indeed, southern Belgium was not an endemic area with respect to the 2012 results.

However, Belgian farmers have continued to import cattle (Table 1 and Table 2) from countries considered at risk, mostly from France. In 2018, a surveillance system was successfully put in place and all imported animals from countries at risk are now tested. Since 2020, this monitoring system has allowed us to identify and trace five infected animals in four different herds (H9, H10, H11, and H12). These imported and infected animals coming from southern departments of France seemed to be asymptomatic carriers according to veterinary practitioners. Despite the advice given and in the absence of regulation, two of the four breeders made the decision to keep the animals despite the risk of the disease spreading. According to them, only significant financial compensation would convince them to slaughter those animals. Appropriate policies should be adopted by authorities. At the same time and while waiting for law enforcement, farmers’ consensus should be enhanced.

Moreover, one year before, in May 2019, the first outbreak herd was detected with animals presenting with clinical signs typical of the chronic phase of the disease. A determination on the exact date of the disease’s introduction into the herd was not possible because the farmer had been purchasing animals from endemic areas for over 10 years. Given the prevalence of almost 19.6% of the animals in that herd and the epidemiology of besnoitiosis according to several studies [5,21,22,23], a likely hypothesis could be that the infection was introduced relatively recently. This would match with the purchase of cattle from Aveyron, a department in France where the disease is endemic. Actually, one bull was imported into the herd on 16 February 2015 (the bull whose symptoms are described in Figure 2). In addition, a cow born in Belgium on 18 January 2017 was also introduced and its mother was imported in March 2010 from France with no clinical signs. Both animals were *B. besnoiti* Ab ELISA and WB positive.

Since the lesions are chronic and therefore several months old, it is not possible to determine the date of infection with precision using those lesions. Whether the disease was introduced in 2015 or in 2010, the proportion of infected animals in the herd is low compared with other conditions in the event of the introduction of the disease. Indeed, when bovine besnoitiosis is introduced, the incidence of the disease is often very high [21]. Numerous studies show that the annual incidence varies from 39.6% in northern Italy [22] to 60.65% in southern France [5] and 36.1% over four months in southwestern Spain [23]. Another study [24] found an intra-herd seroprevalence ranging from 79.2% to 100% in a region newly affected by the parasite. The differences in the Belgian climate could have an effect on the vectors’ activity with consequences for the disease’s incidence. The incidence observed in this study is lower than the ones published in studies carried out in southern European countries.

The disease still had time to be transmitted to a nearby farm due to the pasture’s proximity. Upon investigation of other six contact herds, only one herd was found to be infected (Table 4). In the contact herd infected through a grazing contact, only two animals were seropositive (3.6%). This low prevalence also indicates a recent infection.

In southern Belgium, different species of blood-sucking insects, such as tabanid species and stable flies (*Stomoxys calcitrans*), may transmit *B. besnoiti* mechanically from chronically or asymptomatic infected cattle. The insects responsible for the current transmission in this case are unknown but the relatively small radius of activity of these insects may explain the low spread via pasture contacts [25,26]. In addition, there is a need for close contact between cattle for transmission of bovine besnoitiosis to take place since the vector insects are infectious only within hours of their blood meal on a sick animal. The maximum time following a blood meal on an infected animal during which vectors remain infectious is low and varies according to species. It is one hour in the case of *S. calcitrans*, three hours for *G. brevipalpis*, 24 h for tabanids, and 50 h for *Culex* [6,20].

In this herd, the infection was quickly controlled by serological testing and slaughtering of the infected animals. The farmer understood the risk and the economic stakes associated with this disease and therefore took the necessary action without being obliged to do so.

In order to ensure the control/eradication of bovine besnoitiosis in this commune, ARSIA advised serological monitoring of contact herds over the next 3 years. Regarding the two other herds infected following the purchase of animals from outbreak farms H1 and H2, the infection was also quickly controlled, and the infected animals were culled. Under our conditions, the infected herds presented low seroprevalences. Indeed, the relevant option was to cull all infected animals. This study shows the importance of reacting quickly. The detection of the infection one or more years earlier could have prevented the infection of many animals and consequently their premature culling. More studies are needed to model the spread of the disease in a herd in order to test management options.

For the health of Belgian livestock, the identification of these cases slowed down the spread of the disease to a large number of herds. The number of secondary outbreaks, either on a pasture or through the purchase of animals, was relatively limited. The economic consequences and management of outbreaks are feasible given the small number of herds affected. Some articles have also mentioned the risk of bovine besnoitiosis transmission to wildlife [27]. Indeed, even if the risk seems to be limited [27], it is important, as a precautionary principle, to manage the outbreaks quickly. Thus, diagnostic tools are keys to the successful implementation and monitoring of control programs. In infected herds, the most widely used approach relies on an accurate clinical inspection supplemented by serology of the whole herd [12].

Following the first outbreak and the identification of infected herds through tracing, the active monitoring system identified eight new outbreak farms (from H5 to H12, Table 4). Tracing is currently being carried out in these herds and measures must be taken as quickly as possible to limit the spread of the disease.

However, the lack of a policy makes it difficult to apply proactive measures in the field as everything depends on the farmer’s willingness to eradicate the disease. All infected cattle in the herds (H1 to H4) traced during the first outbreak were slaughtered. Of the eight herds that imported infected animals, only four took the decision to slaughter them quickly. There are therefore still farmers that are aware of the risk but refuse to slaughter infected animals because none of them have shown clinical signs. They are therefore probably asymptomatic infected animals and have nevertheless the potential to transmit the pathogen to other animals in the same or other herds. This kind of infection allows for the insidious introduction and spread of the disease. The suggestion to keep animals in a stable or to graze them on remote pastures is not an appropriate solution. Since the vectors can also be present in a stable, the pathogen can still be transmitted and spread in the population and the best way to mitigate the risk is culling as described by Sagerman and collaborators in 2018 [28]. Currently, none of the eight importing farmers have reported the appearance of clinical signs. Without a policy and concomitant financial interventions for culling by the authorities, it seems that it will be difficult to fight this disease effectively based on voluntarism. For this reason, a working group was set up to rapidly draft a law that would allow animal health decision-makers to effectively manage outbreaks by having the legal framework to require testing and slaughtering of infected cattle from the population. Despites these national initiatives, information sharing between countries and the brainstorming of future European regulations are extremely relevant and needed in order to keep trade between European countries safe.

## 4. Materials and Methods

### 4.1. Study Area

The study was carried out in southern Belgium in a region called Wallonia, one of the two regions of Belgium. The cattle population was approximately 1 million cattle (about one third dairy cattle and two thirds beef cattle). Among the beef cattle, the Belgian blue-white breed was strongly represented; however, many breeders have decided to carry out extensive breeding and to import French breeds.

### 4.2. Sampling

#### 4.2.1. Epidemiological Screening

**Figure 4:**
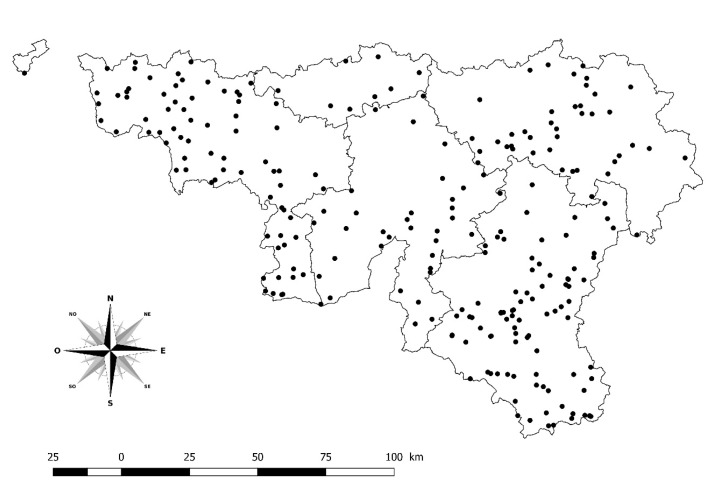
Geographical distribution of screened herds in southern Belgium. Legend: Solid lines display the borders of the five provinces in southern Belgium included in this study (i.e., Hainaut, Brabant Wallon, Liège, Luxembourg, and Namur).

In 2012, in order to monitor the infection in southern Belgium, the laboratory of ARSIA decided to carry out epidemiological screening of 5034 samples selected from a collection of sera between February 2011 and November 2012. These samples were collected from 284 herds (Figure 4) with a history of purchasing cattle from France. Spain and Portugal are also countries where the disease is endemic, but the number of imports from these countries is low and no imports from Spain and Portugal were recorded during that period. The samples used originated from a collection of randomly selected sera of existing monitoring programs (i.e., infectious bovine rhinotracheitis (IBR), paratuberculosis, and brucellosis). Serological screening was performed and about twenty animals over 12 months old per herd were sampled by an epidemiosurveillance veterinarian.

#### 4.2.2. Serological Testing at Importation

As the risk of importation of the disease has been growing since 2018 and following the previous cases of imports into Germany, Italy, and Switzerland, ARSIA decided to systematically control the import of animals from countries considered to be at risk (France, Spain, Portugal, Italy, and Switzerland). Indeed, all imported cattle from these five countries were tested from 2018 to 2021.

#### 4.2.3. Control of All Live Animals Imported from Countries at Risk

After the discovery of the initial infected cases in a herd where imported cattle from France were raised, ARSIA requested the testing of all alive animals, that is, 4077 animals in 895 herds, not yet tested for besnoitiosis and imported from countries at risk. The identity of the animals was added to the lists of animals to be sampled during winter prophylaxis.

#### 4.2.4. Awareness Campaign towards the Breeding Sector

In 2018, ARSIA started an awareness campaign in order to inform veterinarians about the risk and the evocative clinical signs using pictures. When a clinically suspect animal was identified following the tracing back or the tracing forward of an infected animal, serum from this animal was collected by the epidemiological surveillance veterinarian. If the suspicion was confirmed, sampling was performed in herds at risk and consisted of dry tube serum samples from all animals over 6 months of age (in order to avoid interference with colostral immunity).

In total, 138 suspected cases were sampled in 99 different herds. All samples were analysed, but only one herd was confirmed to be infected (the first outbreak, H1). All serum samples from ARSIA were stored at −20 °C until analysed at the regional laboratory.

### 4.3. Epidemiological Inquiry

**Figure 5:**
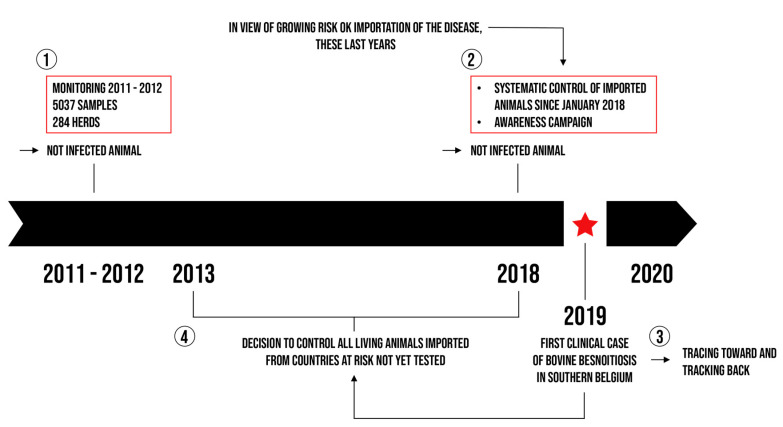
Illustration of the evolution of the epidemiological surveillance in southern Belgium (step 1 to step 4).

The tracing back of the first case was initially carried out by a veterinarian from ARSIA by contacting the GDS of the concerned department in France, the association responsible for animal health in Aveyron where the herd from which the imported cattle came was located. In order to continue tracing back and given the possible involvement of other departments, monitoring was done with the support of GDS France. The latter brings together all the associations responsible for animal health in France (Figure 5).

The tracing forward was performed by veterinarians from ARSIA using SANITEL to identify all contact farms in northern and southern Belgium. Serological screening results were only obtained for southern Belgian herds during the study.

The main risks were grazing contacts and the sale of cattle. Regarding the risk associated with grazing, a farm was part of the tracing if its animals had grazed close to an infected cattle pasture within a distance of 200 m according to previous guidelines [25,26]. Regarding the risk associated with the sale of cattle, a farm was part of the tracing if it purchased cattle from infected farms. The incubation period (usually between 6 and 10 days) [4] was not taken into account because infected animals can represent a risk from their first day of introduction into the herd due to the possible presence of infecting cysts. In addition, some farms were only fattening adult animals or calves. In these kinds of herds, animals were reared for approximately 8 months for meat, did not graze, and were not mated. These farms were not considered to be at risk and no blood samples were taken as part of the tracing. When a farm was part of the tracing, serological screening was carried out and, depending on the situation, included either all animals in the herd, animals older than 6 months, or animals randomly selected.

### 4.4. Clinical Presentation

Upon disease diagnosis by a veterinary practitioner, ARSIA veterinarians paid a visit to the concerned farm to provide advice, perform a clinical examination, confirm the clinical signs, and photograph the infected animals. Information collected was shared with all farmers and local veterinary practitioners aiming at increasing clinical disease detection in the area.

### 4.5. Serological Examination

#### Serological Screening

Once a year, each IBR-free herd has to maintain its status using serological control. A random selection of a certain number of animals is carried out and the number of animals to be sampled (one to 20) depends on the herd’s size. The sampling method used is available in the Royal Decree related to IBR control [29] and is presented in Table 6.

The test routinely used for import and monitoring is indirect ELISA. All ELISA seropositive and doubtful sera were sent to the Ecole nationale vétérinaire de Toulouse (ENVT) and confirmed using the Western Blot technique. Western blotting has been recommended as a confirmatory test when coupled with other methods such as ELISA [30,31,32,33].

ELISA: *B. besnoiti* diagnosis in the serum was performed using an indirect bi-well ELISA for the detection of antibodies against *B. besnoiti* in bovine serum [31] according to the manufacturer’s instructions (The ID Screen^®^ Besnoitia Indirect 2.0, Montpellier, France). Briefly, the wells were sensitised with purified antigenic extract of *B. besnoiti*. Test samples and controls were distributed in odd and even wells. *B. besnoiti* specific antibodies, if present, form an antigen–antibody complex. A peroxidase-labeled anti-ruminant conjugate (HRP) was distributed into the wells. It binds to anti-*Besnoitia* antibodies, forming an antigen–antibody–conjugate–HRP complex. After the excess conjugate was removed by washing, the reaction was revealed by developing solution (TMB). The optical densities of the positive and negative sera (OD pos and OD neg) and those of all the samples (OD samples) were measured. The sample/positive control (S/P) ratio, expressed as a percentage, was calculated as follows: S/P ratio = [(OD sample − OD neg)/(OD pos − OD neg)*100. The interpretation of the results was carried out according to the following magnitude scale: negative (S/P ≤ 25%), doubtful (25% ≤ S/P ≤ 30%), positive (S/P > 30%). According to the producer, the ELISA used has a sensitivity of 98.1% and a specificity of 100%.

Western blot (WB): WB was performed in the Laboratoire National de Contrôle des Reproducteurs (ACSEDIATE/LNCR). Infected Vero cells and free tachyzoites were harvested and centrifuged at 5000 rpm for 10 min at 4 °C. The pellets were washed with PBS, separated on a Sephadex G-25 column (GE Healthcare Europe Gmbh, Ref 17-0851-01, Orsay, France), and then centrifuged at 1200× *g* for 10 min at 4 °C. Pellets of tachyzoites were frozen at −80 °C until use. Pellets were solubilised and denatured by heating at 95 °C for 10 min. Electrophoresis was performed in SDS-PAGE (12.5%, *w/v*) gels using 15 µL of sample/cm of slot. Then, B. besnoiti antigens were electrophoretically transferred to PVDF membranes (Immun-Blot^®^ PVDF Membrane for Protein Blotting, Biorad, Ref 162-0177, Marne La Coquette, France) adapted from [30].

Bovine sera, diluted to 1:50 in PBS, 0.05% (*v/v*) Tween 20, and 2% (*w/v*) milk protein, were incubated for 15 min at room temperature under gentle agitation and then at 37 °C for 45 min with strips previously blocked with skim milk (after incubation for 30 min at room temperature). Strips were washed with PBS + Tween 20. Antibody reactions were highlighted with an anti-bovine IgG (H + L) peroxidase conjugate (Sigma–Aldrich, Ref A 5295, St Quentin Fallavier, France) diluted in PBS + Tween 20 at 1:150 and visualised using the Opti 4CN substrate kit (Biorad, Ref 170-3932, Marne La Coquette, France). Molecular weights of the different antigens recognised by sera were determined by comparison with the respective molecular weight standards (Precision Plus Protein Dual Color Standards, Biorad, Ref 161 0374 S04, Marne La Coquette, France). A serum sample was considered positive when the three main antigenic domains were observed as documented by Cortes and collaborators in 2006: domain I ranging between 12 and 20 kDa; domain II ranging between 23 and 38 kDa; and domain III ranging between 60 and 90 kDa with at least a set of four bands within each of them. This WB technique exhibited a diagnostic sensitivity of 91.3% and a specificity of 96.4% to 100% [30].

## 5. Conclusions

This study is interesting because it is the first evidence of besnoitiosis in southern Belgium. Moreover, it is the first study to highlight cases of transmission through grazing contacts and sales of animals in Belgium. Implementing a rigorous tracing and working methodology is essential and can be improved. In order to ensure the effective eradication of the disease during outbreaks, it will be essential to continue monitoring the exposed population over the next few years and maintain a deep surveillance of cattle imports.

This study allows for a better understanding of the epidemiological situation of southern Belgium with regard to bovine besnoitiosis. Moreover, it highlights the high risk of the introduction of such a disease in the concerned region following imports from areas at risk. Better guidelines are needed to ensure that trade is safe between countries at the European Union level.

This study shows that besnoitioisis-free countries should not limit themselves to passive surveillance of the disease until clinical signs are detected by veterinary practitioners. It is essential to set up a mandatory active surveillance system via a systematic analysis of all imported animals originating from areas at risk.

In view of the management of this first outbreak of besnoitiosis, early detection has to be improved. The lack of control means other than the culling of infected animals makes it essential to react quickly in order to limit the spread and the economic losses. Moreover, given the absence of clinical signs during the import of asymptomatic infected animals, some breeders are less aware of the risk and the consequences linked to this disease. A targeted communication campaign should be organized to raise awareness in the sector in addition to systematic testing procedures upon importation of cattle from countries at risk.

If the appropriate decisions are not made quickly by authorities and if other upcoming outbreaks are not correctly managed, the deteriorating risk situation may become difficult to control and more financial resources will be required in order to eradicate the disease in southern Belgium. We hope that this practical example encourages the authorities to put in place the appropriate preparedness, detection, and response measures to limit the introduction and the spread of this disease.

## Figures and Tables

**Table 1:** Test results of imported live cattle from January 2018 to July 2021.

Year	Nb of Herds Importing Cattle from Areas at Risk	Nb of Imported Cattle Tested with *B. besnoiti* Ab ELISA	Nb of Bovines with Non-Negative *B. besnoiti* Ab ELISA (Confirmed by WB)	Nb of Herds with Non-Negative *B. besnoiti* Ab ELISA (Confirmed by WB)
2018	206	1816	0 (0)	0 (0)
2019	221	1868	0 (0)	0 (0)
2020	236	2441	6 (1)	2 (1)
2021	159	1497	6 (4)	4 (3)
Total	822	7622	12 (5)	6 (4)

Legend: Nb, number; Ab ELISA, antibody enzyme-linked immunosorbent assay; WB, Western blot.

**Table 2:** Summary of the detection of infected herds via the control of animals previously imported from countries at risk.

Year	Nb of Herds Importing Cattle from Areas at Risk	Nb of Imported Cattle Tested with *B. besnoiti* Ab ELISA	Nb of Bovines with Non-Negative *B. besnoiti* Ab ELISA (Confirmed by WB)	Nb of Herds with Non-Negative *B. besnoiti* Ab ELISA (Confirmed by WB)
2019	130	676	1 (1)	1 (1)
2020	546	2785	7 (5)	5 (3)
2021	67	162	0	0
Total	743	3623	8 (6)	6 (4)

Legend: Nb, number; Ab ELISA, antibody enzyme-linked immunosorbent assay; WB, Western blot.

**Table 3:** Distribution of seropositivity rate per age category in the first infected herd (H1).

Age Category	Nb Tested	Nb Seropositive ELISA (Confirmed by WB)	% Positive in *B. besnoiti* Ab ELISA	% Positive in WB *
6–12 months	35	2 (2)	5.7	5.7
1–2 years	14	0 (0)	0	0
2–4 years	18	7 (4)	38.9	22.2
4–6 years	7	3 (0)	42.9	0
6–8 years	12	3 (2)	25	16.7
>8 years	11	4 (2)	36.4	18.2
Total	97	19 (10)	19.6	10.3

Legend: Nb, number; Ab ELISA, antibody enzyme-linked immunosorbent assay; WB, Western blot; * assuming that negative results of ELISA were negative by Western blot (but not tested).

**Table 4:** The origin of herd infections in southern Belgium.

Infected Herd	Tracing Back
H1	Import of animals coming from three different herds of two departments: Aveyron, Cantal
H2	Grazing contact with pasture from H1
H3	Purchase of animals from H1
H4	Purchase of an animal from H2
H5	Import of animals coming from one herd of one department: Arriège
H6	Import of animals coming from one herd of one department: Hautes-Pyrénées
H7	Import of animals coming from one herd of one department: Gironde
H8	Import of animals coming from one herd of one department: Doubs
H9	Import of animals coming from one herd of one department: Allier
H10	Import of animals coming from two herds of two departments: Hautes-Pyrénées, Lot-et-Garonne
H11	Import of animals coming from one herd of one department: Haute-Vienne
H12	Import of animals coming from one herd of one department: Arriège

**Table 5:** Distribution of seropositivity rate per age category in the first infected herd H2.

Age Category	Nb Tested	Nb Seropositive *B. besnoiti* Ab ELISA (WB)	% Positive in *B. besnoiti* Ab ELISA	% Positive in WB
6–12 months	5	0 (0)	0	0
1–2 years	7	0 (0)	0	0
2–4 years	29	2 (2)	6.9	6.9
4–6 years	11	0 (0)	0	0
6–8 years	2	0 (0)	0	0
>8 years	1	0 (0)	0	0
Total	55	2 (2)	3.6	3.6

Legend: Nb, number; Ab ELISA, antibody enzyme-linked immunosorbent assay; WB, Western blot.

**Table 6:** Herd level sampling size used for serological screening with randomly selected animals.

Herd Size	Number of Cattle to Sample
>260	20
80–260	19
40–79	18
20–39	16
16–19	13
14–15	12
13	11
12	10
10–11	9
1–9	All animals

## Data Availability

The data that support the findings of this study are available from the corresponding author upon request.
